# Aeration and Shear Stress Are Critical Process Parameters for the Production of Oncolytic Measles Virus

**DOI:** 10.3389/fbioe.2019.00078

**Published:** 2019-04-17

**Authors:** Tanja A. Grein, Daniel Loewe, Hauke Dieken, Tobias Weidner, Denise Salzig, Peter Czermak

**Affiliations:** ^1^Institute of Bioprocess Engineering and Pharmaceutical Technology, University of Applied Sciences Mittelhessen, Giessen, Germany; ^2^Faculty of Biology and Chemistry, Justus Liebig University, Giessen, Germany; ^3^Project group Bioresources, Fraunhofer Institute for Molecular Biology and Applied Ecology (IME), Giessen, Germany

**Keywords:** cell culture, microcarrier, total particle collision severity, vaccine, stirred tank reactor, serum-free medium, Vero cells

## Abstract

Oncolytic Measles virus is a promising candidate for cancer treatment, but clinical studies have shown that extremely high doses (up to 10^11^ TCID_50_ per dose) are required to effect a cure. Very high titers of the virus must therefore be achieved during production to ensure an adequate supply. We have previously shown that Measles virus can be produced in Vero cells growing on a Cytodex 1 microcarrier in serum-containing medium using a stirred-tank reactor (STR). However, process optimization and further process transfer or scale up requires the identification of critical process parameters, particularly because the use of STRs increases the risk of cell damage and lower product yields due to shear stress. Using a small-scale STR (0.5 L working volume) we found that Measles virus titers are sensitive to agitator-dependent shear, with shear stress ≥0.25 N m^−2^ reducing the titer by more than four orders of magnitude. This effect was observed in both serum-containing and serum-free medium. At this scale, virus of titers up to 10^10^ TCID_50_ mL^−1^ could be achieved with an average shear stress of 0.1 N m^−2^. We also found that the aeration method affected the virus titer. Aeration was necessary to ensure a sufficient oxygen supply to the Vero cells, and CO_2_ was also needed to regulate the pH of the sodium bicarbonate buffer system. Continuous gassing with air and CO_2_ reduced the virus titer by four orders of magnitude compared to head-space aeration. The manufacture of oncolytic Measles virus in a STR can therefore be defined as a shear-sensitive process, but high titers can nevertheless be achieved by keeping shear stress levels below 0.25 N m^−2^ and by avoiding extensive gassing of the medium.

## Introduction

Measles virus has natural oncolytic properties and an excellent safety profile as an attenuated vaccine (WHO, 1998). Engineered oncolytic Measles viruses selectively kill cancer cells and induce a systemic anti-tumor immune response, making this virus an attractive choice for the treatment of patients suffering from incurable cancer (Rammensee, 2014; Russell et al., 2014; Galanis et al., 2015). Measles viruses have been engineered for the treatment of various types of cancer for more than a decade (Peng et al., 2002; Blechacz et al., 2006; Muhlebach et al., 2010; Msaouel et al., 2011; Guillerme et al., 2013; Zhao et al., 2013; Russell et al., 2014).

Clinical studies have shown that high doses of oncolytic Measles virus (10^8^-10^11^ TCID_50_ per dose) are required for successful treatment (Russell et al., 2014; Galanis et al., 2015). High-titer production processes are therefore needed to ensure a sufficient supply of the virus. Current production methods for the measles vaccine do not yield enough virus for oncolytic therapy, with maximum titers of 10^6^ TCID_50_ mL^−1^ reported for mammalian cells growing on microcarriers (Trabelsi et al., 2014). We recently described an adapted process that can achieve titers of 10^10^ TCID_50_ mL^−1^, in which Vero host cells attached to Cytodex 1 microcarriers are cultivated in a small-scale stirred-tank reactor (STR) using serum-containing medium (Grein et al., 2018). The high titers were realized by harvesting the thermosensitive virus at the optimal time, determined online by dielectric spectroscopy (Grein et al., 2018).

The STR is a double-edged sword because the power input needed for homogeneous cell distribution generates shear stress that has the potential to cause cell and virus damage. The system must therefore be optimized to ensure homogeneous cell distribution while limiting shear stress. The shear sensitivity of Measles virus is not well-characterized, but the impact of shear must be understood in detail before process scale-up, or process modification such as switching to serum-free medium, given that serum is known to protect cells against shear stress (Chisti, 2000; Santos et al., 2011). The use of incompletely-defined fetal bovine serum bears the risk of product contamination and variation in product yields, and the regulatory agencies therefore require the use of serum-free medium for the production of biopharmaceuticals, including oncolytic viruses, to reduce risks to patients (Tripartite, 2012).

The effect of shear stress on cell growth has been investigated in detail. Cell growth in a STR is affected if the eddy size is similar to the particle size of the host cell or microcarrier (van der Pol and Tramper, 1998; Chisti, 2001). The relationship is described by the Kolmogorov model, which gives a correlation between the size of the smallest possible eddy (λ), the energy dissipation in a bioreactor (ε) with volume (V) and the kinematic viscosity of the suspension (υ) as shown in Equations (1,2) (Zlokarnik, 2008).

(1) $$\begin{array}{rcl} \lambda & = & {\left(\frac{\nu^{3}}{\varepsilon }\right)}^{0.25} \end{array}$$

(2) $$\begin{array}{rcl} \varepsilon & = & \frac{P}{\rho^{V}} \end{array}$$

The stirrer-related power input (*P*) can be calculated from the density (ρ) of the solution, the agitation rate (n), the impeller diameter (d_st_) and the dimensionless power number (N_P_) as shown in Equation (3).

(3) $$\begin{array}{r} \text{P}={\text{N}}_{\text{P}}\cdot \rho \cdot {\text{n}}^{3}\cdot {{\text{d}}_{\text{st}}}^{5} \end{array}$$

The average shear stress τ can then be calculated from P and its dynamic viscosity (*v*) as shown in Equation (4).

(4) $$\begin{array}{r} \tau =\eta \cdot {\left(\frac{\varepsilon }{\nu }\right)}^{0.5} \end{array}$$

The equations set out above are true for turbulent flow regimes. Information about the flow regime gives the Reynolds number, as shown in Equation (5).

(5) $$\text{Re=}\frac{\rho \;\cdot \text{ n }\cdot \;{\left({\text{d}}_{\text{st}}\right)}^{\text{2}}}{\eta }$$

Turbulence occurs when Re >10^4^ for non-baffled vessels and when Re >50 for baffled vessels (Doran, 1995; Zlokarnik, 2008). Adherent growing Vero cells tolerate maximum shear stress levels in the range 3.5–5 N m^−2^ (Crouch et al., 1985; Hjortso, 1994), depending on the cultivation device and/or cultivation conditions. Vero cells become less adherent during Measles virus infection (Grein et al., 2018). In contrast to adherent cells, suspended cells can relax tensile and shear stress because they have a higher degree of freedom (Rentier et al., 1978; Ludlow et al., 2013). This may allow the infected cells to tolerate higher levels of stress during Measles virus production, which would explain the wide range in tolerance.

Collisions between microcarriers can also inhibit cell growth and virus production. This effect is not included in the Kolmogorov model, so a factor defined as turbulent collision severity (TCS) has been proposed to describe this negative effect on cell growth, and is the product of the kinetic energy released during a collision between two particles and the collision frequency (Cherry and Papoutsakis, 1988, 1989). The mass (m_mc_) and diameter of the microcarrier (d_mc_), the volume fraction of the microcarrier/solid phase (φ), and their relative velocity (v) define the severity of microcarrier collision, as shown in Equation (6).

(6) $$\begin{array}{r} \text{TCS}=\left(\frac{{\text{m}}_{\text{mc}}\text{ v}}{2}\right)\;\cdot \frac{\left(\frac{\text{v }\phi^{2}}{{\text{d}}^{2}}\right)}{\left(\frac{\phi }{\left(\frac{\pi }{6}\right)\;{\text{d}}_{\text{m}c}^{3}}\right)} \end{array}$$

**Figure 1:**
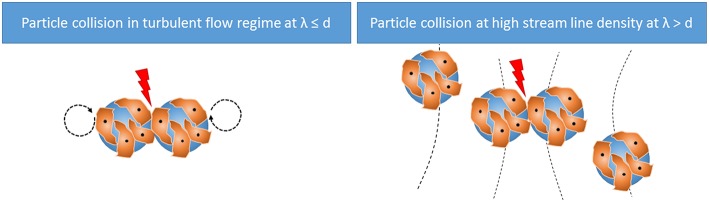
Cell damaging effects according to the theory of Cherry and Papoutsakis.

The relative velocity of the colliding microcarriers (*v*) can be estimated using two methods (Figure 1). If λ ≤ d_mc_, the velocity of the microcarrier is assumed to be in the same range as the Kolmogorov eddies. Therefore, the TCS in this case is dominated by these eddies (TCS_ε_) as shown in Equation (7).

(7) $$\begin{array}{r} TCS_{\varepsilon }={\left(\varepsilon \;\upsilon \right)}^{3/4}\;\left(\frac{\pi^{2}\;\rho_{mc}\;d_{mc}^{2}\;\phi }{72}\right) \end{array}$$

In the presence of eddies larger than the microcarrier size (λ > d_mc_), the microcarriers align along the streamlines. Here, the distance between the microcarriers is the distance of the streamlines. If the streamline density is too high, particle collision will occur even though eddies would be too large to shear the microcarriers directly.

Accordingly, in turbulent flow regimes where λ > d_mc_, the velocity of the microcarrier can be estimated from the velocity gradient or the shear rate. Therefore, the TCS in this case is dominated by the streamlines (TCS_s_) as shown in Equation (8).

(8) $$\begin{array}{r} TCS_{s}={\left(\frac{\varepsilon }{\upsilon }\right)}^{2/3}\;\left(\frac{\pi^{2}\;\rho_{mc}\;d_{mc}^{5}\;\phi }{72}\right) \end{array}$$

It is unclear whether Kolmogorov-related shear stress or the TCS of the microcarrier (or both phenomena) are responsible for reducing Measles virus titers in a STR.

Cell growth can also be affected by bubble aeration (Chisti, 2001; Quesney et al., 2003). In mammalian cell cultures, aeration is needed to supply oxygen, and CO_2_ is needed if a sodium bicarbonate buffer is used to regulate the pH of the cell culture medium. The mechanisms responsible for cell damage due to mixing and aeration have been described in detail (Rewatkar et al., 1991; Flickinger et al., 2010).

In order to achieve a reliable supply of oncolytic Measles virus for cancer patients while fulfilling all regulatory demands, we characterized the shear sensitivity of the Measles virus production process in a STR, focusing on the effects of shear stress caused by agitation and gassing, and also comparing the effect of these parameters on cells growing in serum-containing and serum-free media.

## Materials and Methods

### Host Cell Line and Virus Strain

The Measles virus strain MVvac2 GFP (P) #312 was propagated in Vero cells (#CCL-81, ATCC) as previously described (Devaux et al., 2007). MVvac2 GFP (P) is an infectious recombinant vaccine strain, which was kindly provided by Dr. Michael Muehlebach (Paul-Ehrlich-Institute, Langen, Germany). To ensure consistent production conditions in the STR, we used Vero host cells with the same passage number (P170–P172), which were infected with an aliquot from the same Measles virus stock (#312) with a multiplicity of infection (MOI) of 30 TCID_50_ per cell. The MOI was verified by the determination of the infective dose (TCID_50_, see section Analytics) and the cell concentration at time of infection. Here a sample of the virus stock solution and the cell inoculum were taken before the Measles virus production in the STR was started. The host cells were infected 4–6 h after inoculation by adding the virus suspension. The cell growth was estimated based on the determined cell concentration (c_c_) at process time (t_i_).

(9) $$\text{u}\left(\text{t}\right)\text{ = }\frac{\left(\ln\left({\text{c}}_{\text{c},\text{2}}\right)\;-\;\ln\left({\text{c}}_{\text{c},\text{1}}\right)\right)}{\left({\text{t}}_{2}\;-\;{\text{t}}_{1}\right)}$$

However, host cells have shown maximal cytopathic effect and cell lysis at t > 60 h p.I. (Grein et al., 2018). As consequence, growth of infected cells was analyzed for the time interval 0–60 h p.I.

### Cell Growth in Serum-Containing Medium

Vero cells were cultivated mainly in DMEM-HG supplemented with 10% (v/v) fetal bovine serum 20 mM HEPES and 4 mM L-glutamine (media and supplements supplied by Biochrom). The seeding train was established in 75–175 cm^2^ T-flasks (Sarstedt). Cells were seeded into T-flasks at an initial cell density of 5 × 10^3^ cm^−2^ and were grown to confluence (80–90%). The cells were detached by washing with 0.15 mL cm^−2^ phosphate-buffered saline (PBS, Biochrom) and then incubating with 0.02 mL cm^−2^ 0.25% (w/v) trypsin (Biochrom) for 8 min at 37°C. The detached cells were resuspended in the same culture medium and centrifuged for 5 min at 300 × g (Megafuge X1R, Heraeus). The cell pellet was resuspended in fresh culture medium and plated in new T-flasks or used for bioreactor inoculation.

### Cell Growth in Serum-Free Medium

Vero cells were adapted to grow in serum-free medium VP-SFM (ThermoFisher Scientific) by increasing the VP-SFM content in four steps (25, 50, 75, and 100%). Vero cells growing in serum-free medium therefore had a higher passage number (P174) than the cells in serum-containing medium. The cells were detached and re-suspended using the procedure described above for cells in serum-containing medium, except the trypsinization step lasted only 5 min and was stopped by adding 0.02 mL cm^−2^ trypsin inhibitor (Sigma-Aldrich).

### Bioreactor Settings

Vero cells and viruses were produced in a 1-L STR (model number Z611000110; Applikon) with a working volume of 0.5 L. Fermentations were carried out in serum-containing and serum-free media. The dished-bottom glass vessel (inner diameter 9.5 cm) was equipped with a vortex marine impeller with a diameter of 45 cm (Z81314RC02, Applikon) and two baffles. Microcarrier were prepared in accordance to the manufactures instruction (also see Supplementary Material). The microcarrier concentration was 3 g L^−1^ Cytodex 1 (GE Healthcare) and the cells were cultivated at 32°C. Mixing time experiments were carried out at 32°C using the phenolphthalein pH shift color change method (Grein et al., 2016). Different levels of shear stress were applied as shown in Table 1, and each experiment was conducted in duplicate.

**Table 1:** Process parameters used to investigate the effect of shear stress on Measles virus titers (calculated in accordance to Equations 1–5).

**Agitation rate (rpm)**	**Reynolds number**	**Rate of energy dissipation per unit mass (m^**−2**^ s^**−3**^)**	**Kolmogorov eddy size [μm]**	**Shear stress (N m^**−2**^)**
50	704	0.004	218	0.060
70	1379	0.010	170	0.099
90	2280	0.021	140	0.144
110	3406	0.037	121	0.194
130	4757	0.062	107	0.250
150	6334	0.095	96	0.309
170	8135	0.138	87	0.373
190	10162	0.193	80	0.441

Glass vessels were siliconized before use. The pH was set to 7.4 ± 0.1 and was regulated by the addition of 1 M NaOH and CO_2_ aeration. The dissolved oxygen concentration was maintained at >50% and measured by an optical DO_2_ probe (Oxy-4 mini, PreSense).

Measles virus production were carried out at a low host cell seeding density of 5,000 cells per cm^−2^ (corresponds to 6.9 × 10^4^ cells mL^−1^) and high host cell concentration of 40,000 cells per cm^2^ (correspond to 5.5 × 10^5^ cells mL^−1^ At low cell concentration the maximum overall aeration time could be limited to 5 min per 5 days at a aeration rate and gas stream of 0.02 vvm. An L-sparger was installed under the impeller to supply oxygen and to allow pH control by CO_2_ aeration. The L-sparger has 7 hollows with a size of 1 mm which are arrange in a row of a length of 1.6 cm. The distance between the hollows and the center of the impeller or the tip of the impeller blades was 3 cm, or 1.9 cm, respectively. The oxygen concentration was measured using a noninvasive PreSens oxygen sensor (Precision Sensing GmbH) and the pH was measured using an Applisens (Applikon) or EasyFerm Bio (Hamilton) pH sensor. The whole process was controlled by a flexible control system built up by Pablo Pino Grace based on a Labmanager (Hightec Zhang). The time of infection (TOI) and time of harvest (TOH) were determined by dielectric spectroscopy (Grein et al., 2018).

### Analytics

The cell concentration was estimated off-line based on cell lysis and nuclear staining using crystal violet (Weber et al., 2007). Glucose and lactate concentrations in the cell-free supernatant were measured using a Biosen C-line analyzer (EKF Diagnostics) according to the manufacturer's instructions. The virus titer was determined 7 days post-infection using the TCID_50_ method (Kärber, 1931; Reed and Muench, 1938).

Measles virus production under constant process parameter was conducted in duplicate. This did not allow a statistical analysis to validate the data. However, for the estimation of the accuracy of the data, the relative error (f) of the used methods were estimated by the calculation of the average ($\bar{N}$) and the standard deviation ($\bar{S}$).

(10) $$\begin{array}{r} \bar{\text{N}}=\frac{1}{x}\sum_{i=0}^{x}\left(N_{i}\right) \end{array}$$

(11) $$\bar{s}=\sqrt{\frac{{\left(N_{i}-\;\bar{N}\right)}^{2}}{\left(x-1\right)}}$$

(12) $$\begin{array}{r} {\bar{f}}_{\text{R}}=\frac{\bar{s}}{\bar{N}}\;\cdot 100\% \end{array}$$

The estimated relative errors are shown in Table 2.

**Table 2:** Estimated relative errors of used measurement methods.

	**Measurement range**	**Sample size (x)**	**Relative error (%)**
Glucose concentration (g L^−1^)	0.1-9	8	1.6
Lactate concentration (g L^−1^)	0.05-3.6	8	3.1
Infective dose (TCID_50_ mL^−1^)	10^1^-10^12^	5	32.7
Cell quantification using crystal-violet (cells mL^−1^)	10^5^-10^7^	5	38.4

## Results

### Measles Virus Production in Serum-Containing Medium Is Influenced by Agitation

**Figure 2:**
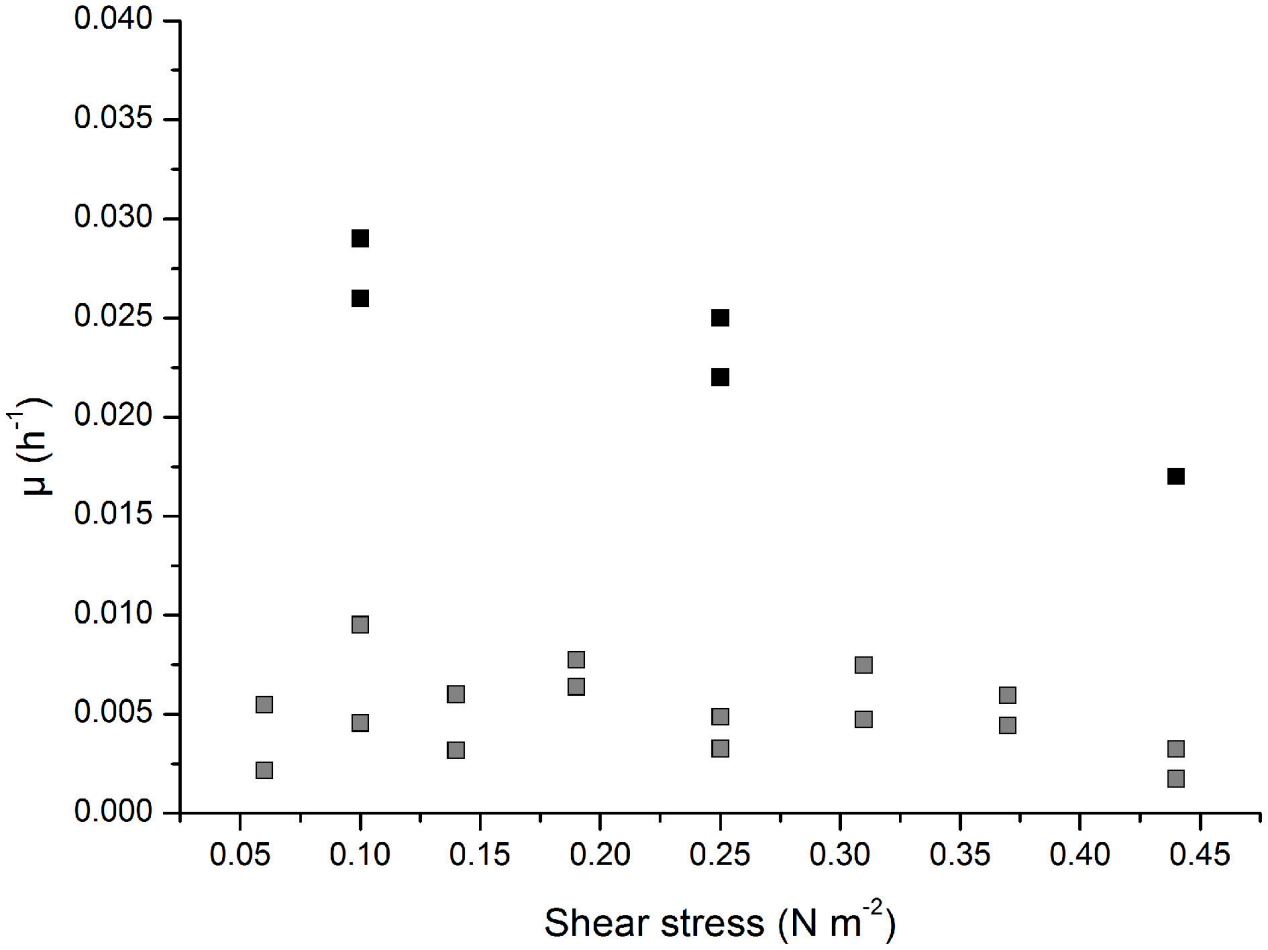
Growth characteristic of infected (gray) and non-infected (black) of Vero cells growing adherent on Cytodex 1 microcarrier in a 0.5 L stirred tank reactor.

The growth characteristics of infected and non-infected Vero host cells growing on the Cytodex 1 microcarrier were determined at different agitation rates in serum-containing medium. As shown in Figure 2, the growth rate of the non-infected cells was 0.029 h^−1^ at the lowest agitation rate of 70 rpm (corresponding to 0.1 N m^−2^) and 0.025 h^−1^ at the moderate rate of 130 rpm (0.25 N m^−2^), but fell to 0.017 h^−1^ at the highest agitation rate of 190 rpm (0.44 N m^−2^). The growth rate of the infected cells was 10-fold lower than the non-infected cells (0.005 ± 0.002 h^−1^ at all agitation rates). This was close to the detection limit, making it impossible to measure the growth rate and the influence of the applied shear stress accurately.

**Figure 3:**
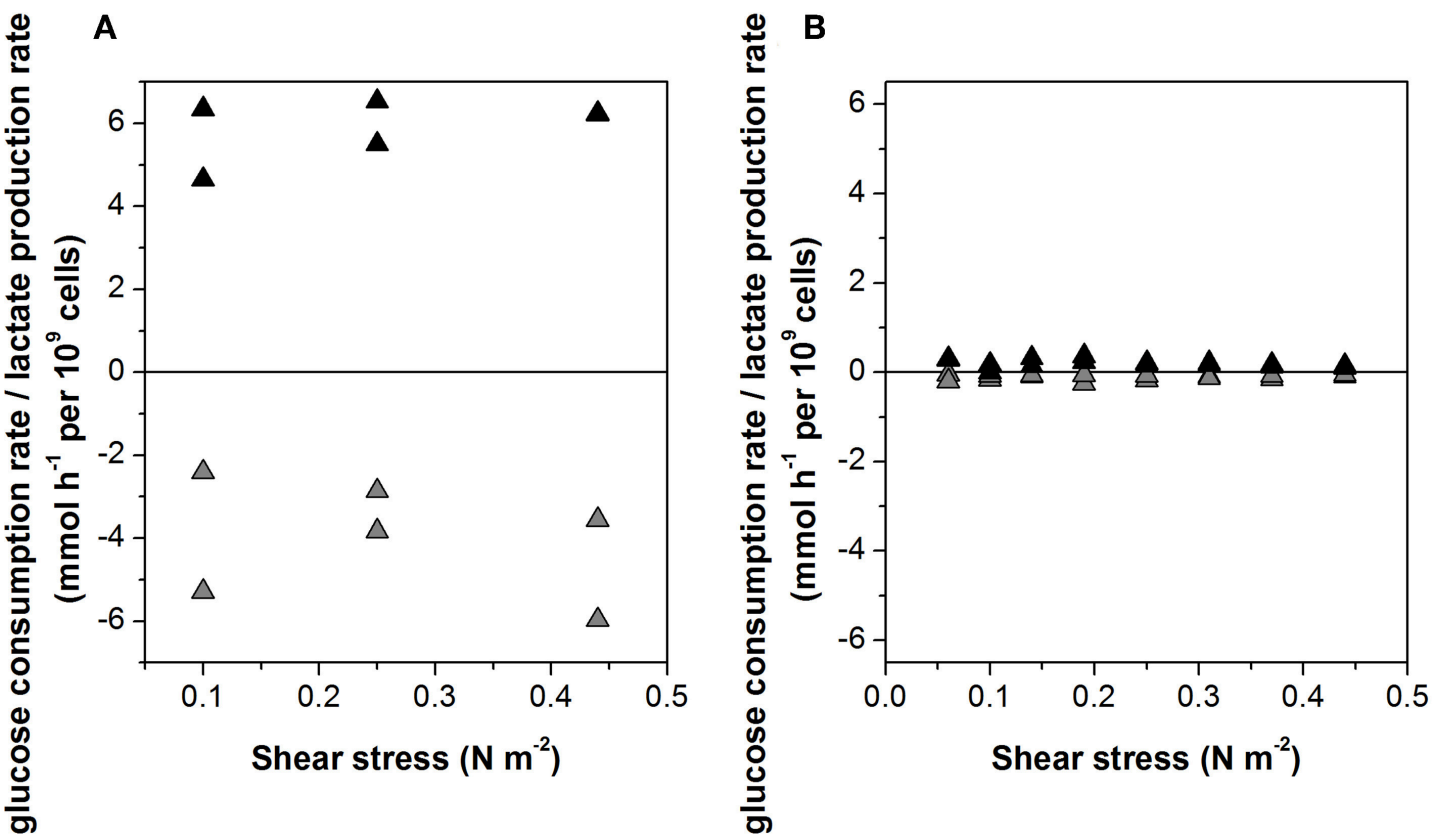
Glucose consumption rate (gray) and lactate production rate (black) of **(A)** non-infected and **(B)** Measles virus infected Vero cells growing on Cytodex 1 microcarrier in a STR.

The specific glucose consumption rate (4.0 ± 1.4 mmol h^−1^ per 10^9^ cells) and the lactate production rate (5.5 ± 1.0 mmol h^−1^ per 10^9^ cells) of the non-infected cells did not change when the agitation rate was increased (Figure 3A). Given the much slower growth rate of the infected cells, glucose consumption and lactate production were also 10-fold lower, at 0.4 ± 0.14 and 0.6 ± 0.10 mmol h^−1^ per 10^9^ cells, respectively (Figure 3B).

**Figure 4:**
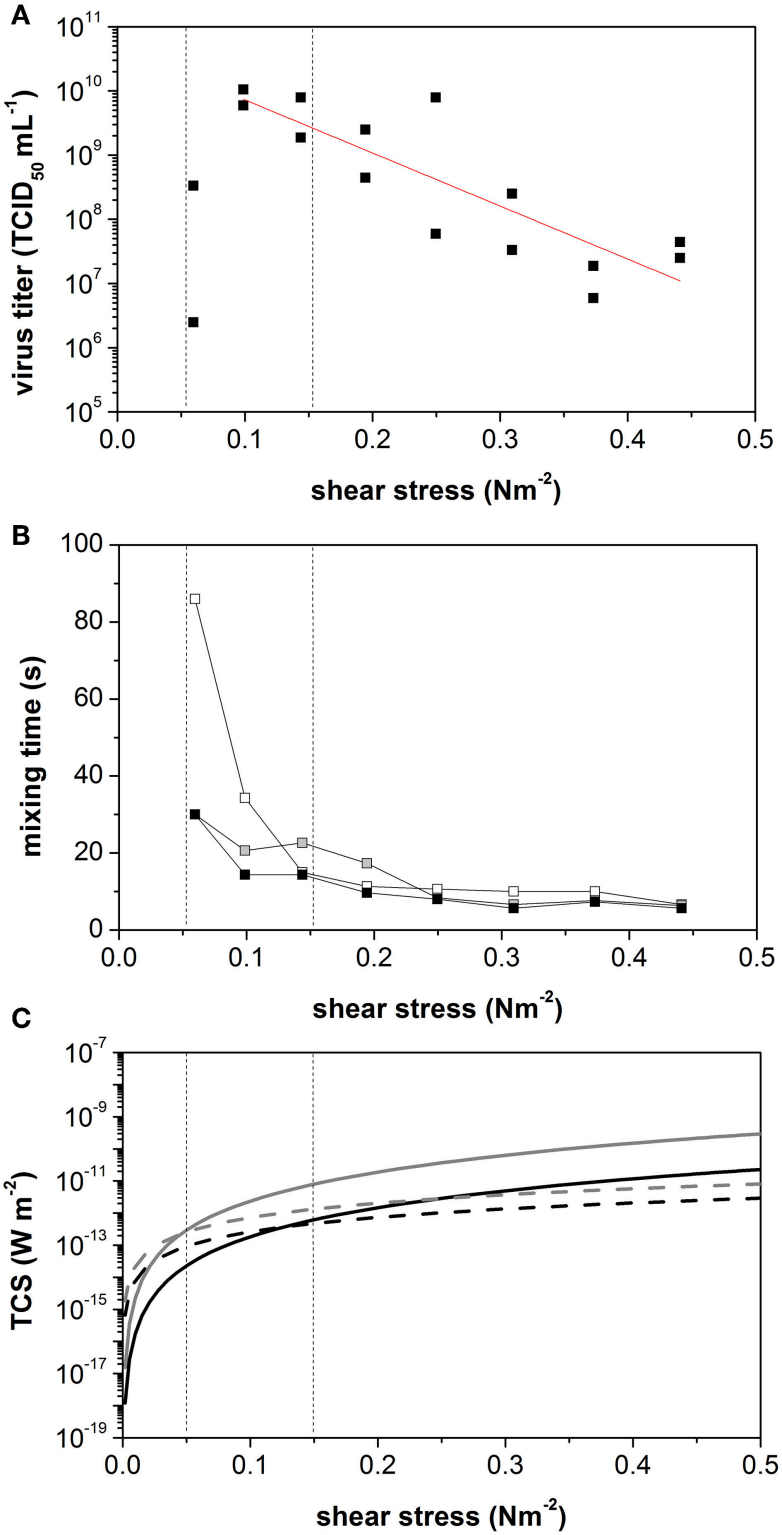
Identification of the dominating effect on measles virus yield produced in vero cells growing on cytodex I in a STR. **(A)** Maximum virus titer during measles production at constant shear stress level in STR. **(B)** Mixing times without microcarrier (white), with 3 g L^−1^ with aeration (black) and without aeration (gray). **(C)** Calculated turbulent collision severity TCS+ (- - Linie) und TCSs (— Linie) for small microcarrier of 150 μm (black) und large microcarrier of 250 μ (gray). Vertical lines were added to indicate the point of intersection of TCD+ and TCSs of small and large microcarrier.

As the agitation rate increased, the maximum virus titer declined from 1.0 × 10^10^ TCID_50_ mL^−1^ to 2.5 × 10^6^ TCID_50_ mL^−1^ reflecting an increase in the average shear stress level from 0.1 to 0.44 N m^−2^ (Figure 4A). The slope of this decline allowed us to calculate a shear stress-related virus titer penalty of −8.3 log_10_ TCID_50_ N^−1^ m^2^. Interestingly, low virus titers were also observed at the lowest tested shear stress of 0.06 N m^−2^, which was caused by the inefficient suspension of the microcarrier under these conditions. In a two-phase system consisting of liquid medium and 3 g L^−1^ Cytodex 1, the minimal mixing time for the suspension was achieved at >90 rpm (Figure 4B). However, at agitation rates in the range 50–90 rpm, the mixing time stagnated. Compared to mixing times established with water alone, we observed a clear influence of the solid component/microcarrier on mixing behavior. In pure water, the mixing time decreased with increasing agitation rate or power input. The minimum mixing time of (6.5 ± 0.5) s was reached at an agitation rate of >70 rpm (0.1 N m^−2^).

These results showed that the microcarriers, as the solid fraction in the suspension, have a major influence on the hydrodynamic behavior of the suspension in the STR. The TCS was therefore calculated to quantify the influence of the microcarrier in the STR. The microcarrier particles are 150–250 μm in diameter. Both, TCS_ε_ and TCS_s_ were calculated for the minimum and maximum microcarrier sizes, using Equations (7) and (8) (Figure 4C). When the calculated TCS data were plotted against τ, the TCS_ε_ and TCS_s_ curves intersected at two points, depending on the microcarrier diameter. The intersect point was at τ = 0.04 N m^−2^ (41 rpm) for the 150-μm particles, and at τ = 0.13 N m^−2^ (82 rpm) for the 250-μm particles. Between these two agitation speeds, we observed the mixing-time plateau, which could be detected in the mixing-time experiments using the two-phase system. If TCS_s_ dominates, enough energy is introduced into the system to achieve the full suspension of the microcarriers. Interestingly, the maximum achievable virus titer also occurs between these two agitation speeds.

### Effect of Aeration on Measles Virus Titers

**Figure 5:**
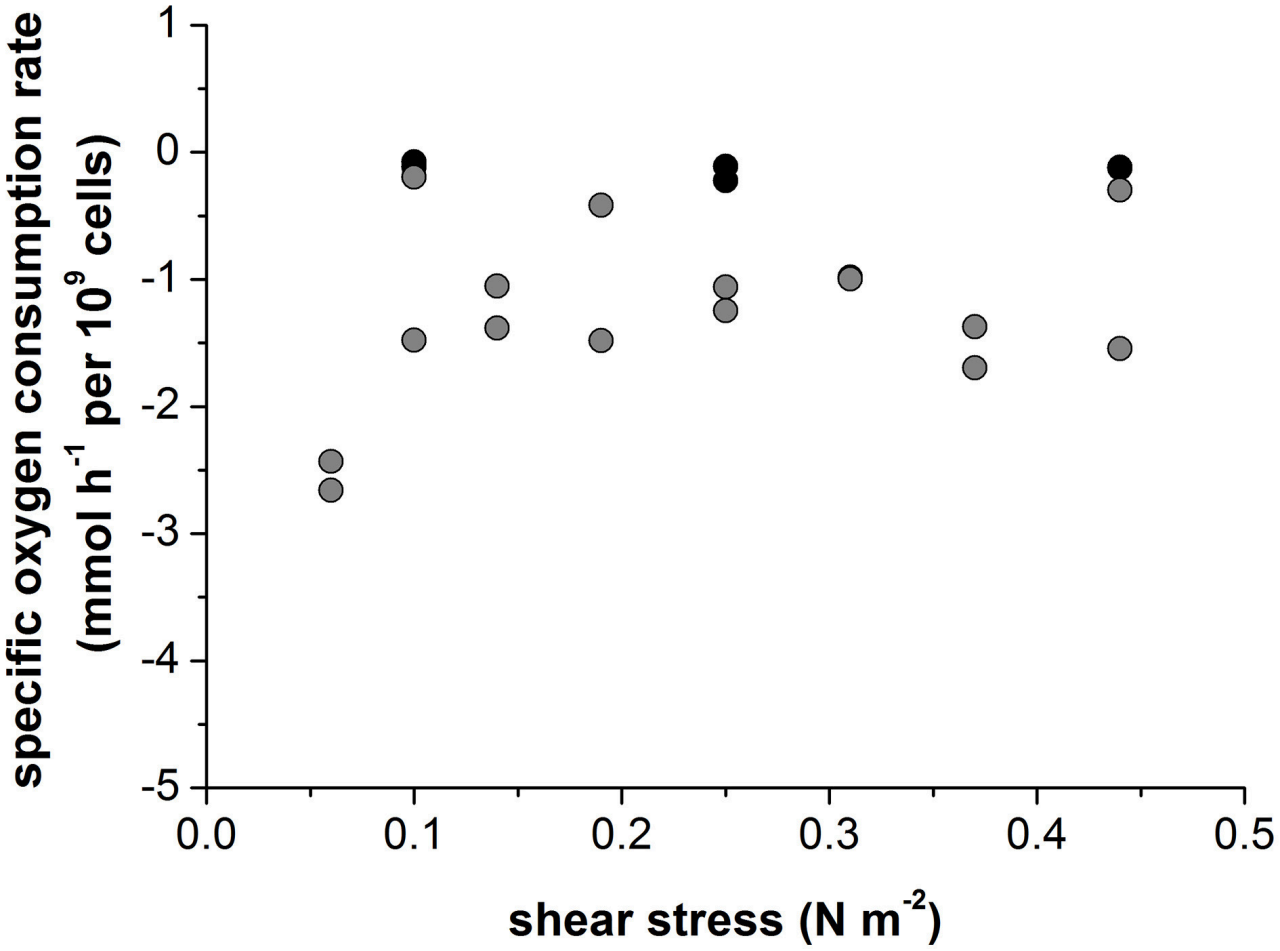
In a 0.5 L STR using Vero cells growing on Cytodex1 microcarrier in serum containing medium. Black: non-infected vero cells; Gray: Measles virus infected Vero cells.

**Figure 6:**
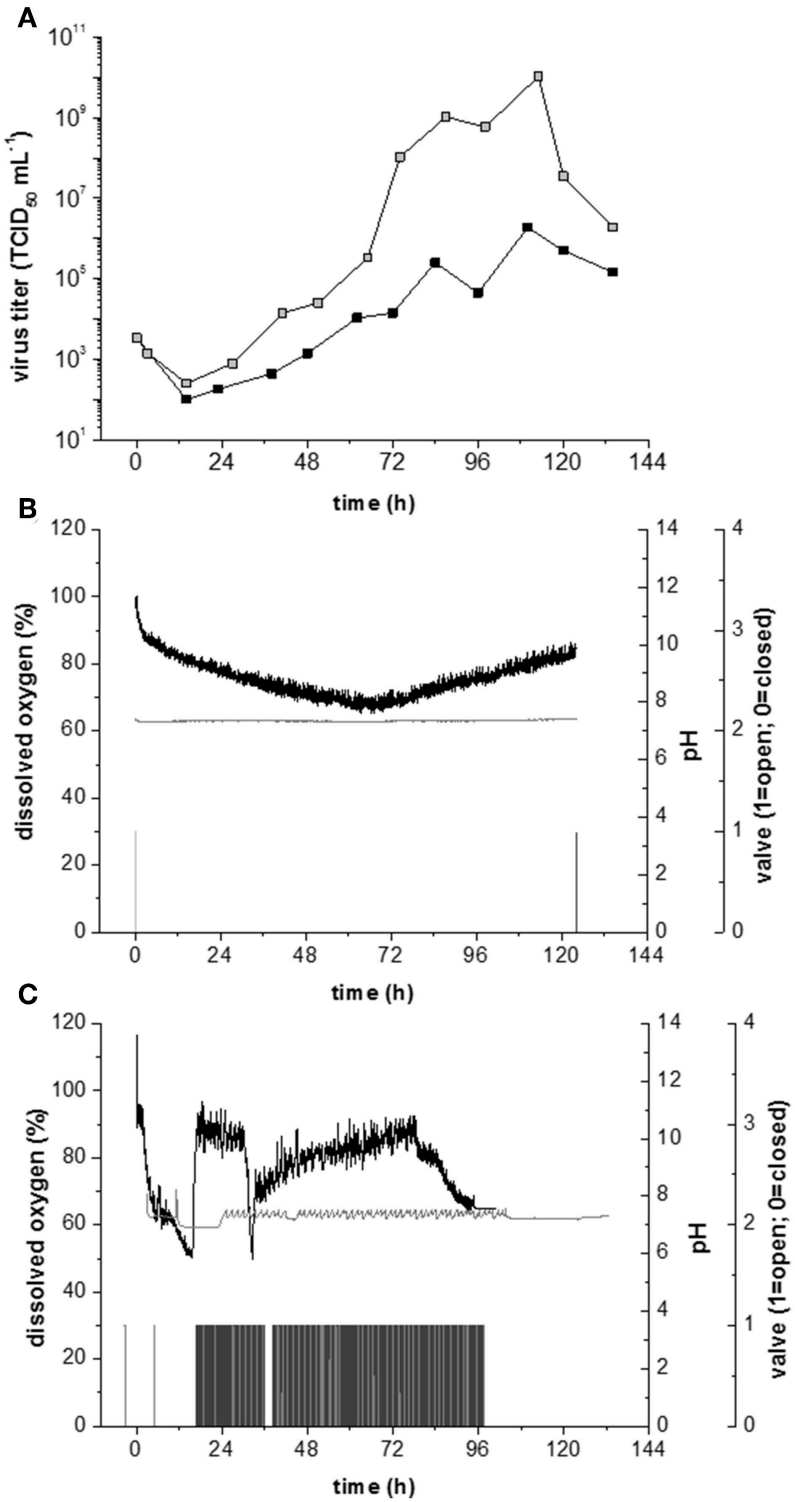
Measles production in Vero cells growing on Cytodex 1 microcarrier at a shear stress level of 0.1 N m^−2^. **(A)** Measles virus titers at continuous gassing (black) and minimum gassing (gray). **(B)** Process parameter during measles production with head space aeration and an inoculation cell concentration of 5,000 cells cm^−2^. Black: dissolved oxygen; Gray: pH value; light gray: Valve of O_2_ aeration; dark gray: Valve of CO_2_ aeration whereby valve 0 = off and 1 = on. **(C)** Measles virus production under continuous aeration; pH value; light gray: Valve of O_2_ aeration; dark gray: Valve of CO_2_ aeration whereby valve value 0 = off and 1 = on.

The non-infected Vero cells growing in serum-containing medium consumed oxygen at a rate of −0.13 ± 0.05 mmol h^−1^ per 10^9^ cells, which was independent of the intensity of shear stress (Figure 5). Infection with Measles virus increased the oxygen consumption rate by 10-fold, to −1.3 ± 0.7 mmol h^−1^ per 10^9^ cells (Figure 5). The high specific oxygen demand of infected Vero cells therefore requires an efficient oxygen supply in the bioreactor. However, continuous aeration at a rate of 0.02 vvm, with otherwise unchanged conditions, reduced the maximum virus titer by four orders of magnitude, to 3 × 10^6^ TCID_50_ mL^−1^ (Figure 6A). In contrast, the maximum virus titer achieved with head-space aeration alone was 10^10^ TCID_50_ mL^−1^ (Figure 6A). The aeration method made no difference to the specific glucose consumption rate (at 0.40 and 0.36 mmol h^−1^ per 10^9^ cells) or the specific lactate production rate (at 0.57 and 0.65 mmol h^−1^ per 10^9^ cells). These results indicated that the active aeration of Vero cells in a STR should be avoided during Measles virus production.

### Effect of High Cell Concentrations at the TOI

In the experiments described above, the density of host cells at the TOI was 5,000 cm^−2^. Increasing the number of host cells to 40.000 cells cm^−2^ at the TOI should achieve higher virus titers. The higher cell density at the TOI had no effect on the growth rate (0.023 h^−1^), specific glucose consumption rate (0.5 × 10^9^ mmol cell^−1^ h^−1^) or specific lactate production rate (0.6 × 10^9^ mmol cell^−1^ h^−1^). However, the high cell density reduced the maximum virus titer by four orders of magnitude to 1 × 10^6^ TCID_50_ mL^−1^. This counterintuitive outcome is likely to reflect the higher oxygen demand of the denser cell population, which can no longer be met by head-space aeration alone (Figure 6B). The higher cell concentrations required an intense aeration strategy to meet the overall oxygen demand (Figure 6C).

In addition to its impact on the maximum virus titer, aeration also affects the pH of the growth medium. Most cell culture media are based on a sodium bicarbonate buffer, allowing the pH to be controlled by the addition of base and gassing with CO_2_. During Measles virus production, the pH usually falls due to lactate accumulation, and the addition of base is sufficient for pH control. However, the intense aeration required for high-density cell populations strips the CO_2_ and increases the pH, so an external CO_2_ supply is needed to maintain a constant pH. Accordingly, we observed a decrease in dissolved oxygen levels because of O_2_ stripping and the DO_2_/pH controller system began to oscillate (Figure 6C). Although this fluctuation was not sufficient to damage the cells, the virus titer was negatively affected, highlighting the importance of appropriate strategies to monitor and control the critical process parameters.

### Measles Virus Production in Serum-Free Medium

Serum-free medium is required by regulatory agencies for the preparation of gene therapy medicinal products (GTMP) for clinical applications, but this makes host cells more sensitive to shear stress and we have already demonstrated that the Measles virus titers are strongly influenced by shear stress in the STR. We therefore compared the performance of Vero cells during expansion in serum-containing and serum-free media, at low (0.1 N m^−2^), moderate (0.25 N m^−2^) and high (0.44 N m^−2^) shear rates. Measles virus production was initiated at a low cell concentration (5,000 cm^−2^) with head-space aeration alone to avoid the negative effects of gassing.

The growth of non-infected Vero cells in serum-free medium was dependent on shear stress. The cell growth rate declined from 0.023 h^−1^ at 0.1 N m^−2^, to 0.017 h^−1^ at 0.25 N m^−2^, and to 0.004 h^−1^ at 0.44 N m^−2^. The specific glucose consumption rates under low and moderate shear stress conditions were −3.4 and −3.9 mmol h^−1^ per 10^9^ cells, similar to the values observed in serum-containing medium. Likewise, the specific lactate production rates under low and moderate shear stress conditions were 4.8 and 5.0 mmol h^−1^ per 10^9^ cells, also similar to the cells in serum-containing medium. With high shear stress, both the glucose consumption and lactate production of the Vero cells in serum-free medium fell below the detection limit.

**Figure 7:**
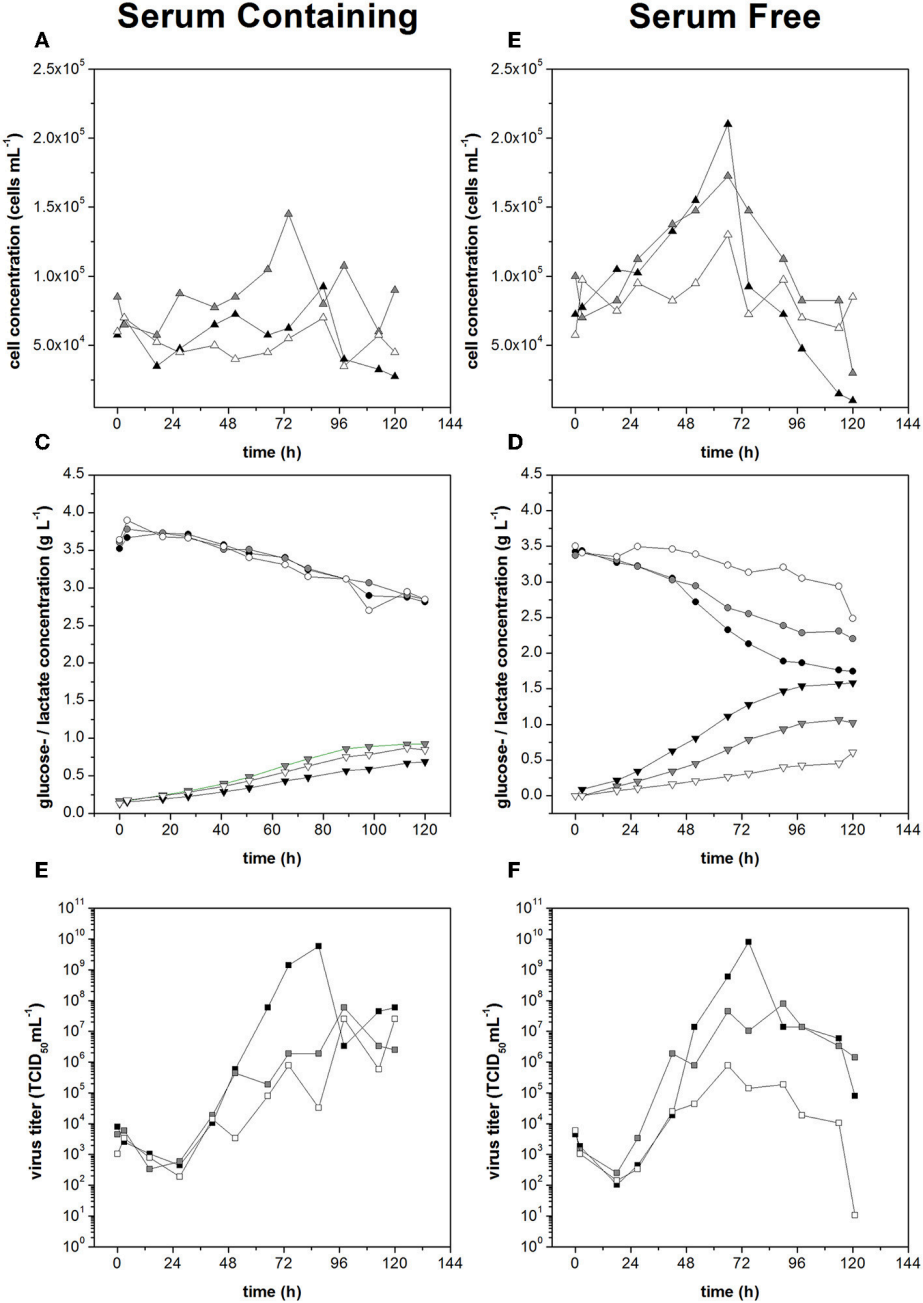
Cell concentration during measles virus production in serum containing medium **(A)** and serum free medium **(B)**. Glucose consumption (•) and lactate production (▴) during Measles virus production in Vero cells using **(C)** serum containing medium and **(D)** serum free medium. Virus titer during measles virus production in serum containing **(E)** and serum free medium **(F)** at different applied shear stress levels (black: 0.1 N m^−2^, gray: 0.25 N m^−2^, white: 0.44 N m^−2^).

The infected cells showed no sign of growth in the serum-containing medium, regardless of the shear stress level. In contrast, the infected cells in the serum-free medium achieved growth rates of 0.010 h^−1^ under low shear stress and 0.009 h^−1^ under moderate shear stress, although only 0.003 h^−1^ under high shear stress, the latter presumably representing growth arrest (Figures 7A,B). The higher growth rates in serum-free medium resulted in a higher glucose demand and a higher rate of lactate production, with the glucose demand showing evidence of shear stress dependence (Figures 7C,D). Comparable specific glucose consumption rates of 0.63 and 0.68 mmol h^−1^ per 10^9^ cells were observed under low and moderate shear stress, but this fell to 3.7 mmol h^−1^ per 10^9^ cells under high shear stress. The lactate production rate remained at 3.8–4.0 mmol h^−1^ per 1^−9^ cells regardless of the shear stress level.

The maximum virus titers under low and moderate levels of shear stress were the same in both media (7.9 × 10^9^ TCID_50_ mL^−1^) under low shear stress and 7.9 × 10^8^ TCID_50_ mL^−1^ under moderate shear stress. When the shear stress was high, we observed a serum-dependent protective effect, with titers of 3.5 × 10^7^ TCID_50_ mL^−1^ in serum containing medium and 7.9 × 10^5^ TCID_50_ mL^−1^ in serum-free medium, two orders of magnitude lower (Figures 7E,F). Therefore, if shear stress levels ≥0.25 N m^−2^ were avoided in the STR, serum-free and serum-containing media achieved the same maximum Measles virus titers.

## Discussion

The growth of Vero cells in suspension is inhibited when shear stress levels exceed 3.5 N m^−2^ (Crouch et al., 1985; Hjortso, 1994). However, we found that adherent Vero cells growing on Cytodex 1 microcarriers in a STR were negatively affected by much lower levels of shear stress (>0.25 N m^−2^). Cells in a shear field suffer damage if the minimum eddy size λ ≤ d_c_ (Kolmogorov et al., 1941; Henzler and Biedermann, 1996). For suspended cells, d_c_ is the cell diameter, but for adherent cells growing on microcarriers, d_c_ is the diameter of the cell–microcarrier construct. The growth of adherent mammalian cells is affected when λ is two thirds of the microcarrier diameter, d_mc_ (Croughan et al., 1989). Cytodex 1 microcarriers range in diameter from 150 to 250 μm, suggesting that cell growth would begin to be affected when reaches λ ≤ 2/3 d_mc_ (100–167 μm). In our STR system, such eddy sizes were created at agitation rates of 79–168 rpm, equivalent to shear stress levels in the range 0.12–0.37 N m^−2^ (mean 0.25 N m^−2^). As predicted by Croughan et al. (1989), our results confirmed that Vero cell growth and Measles virus production were affected under these conditions. Given the calculated TCS, this arbitrary size of two thirds of the diameter of a microcarrier can be explained by the fluid dynamics in the bioreactor. At shear stress levels ≥0.25 N m^−2^, the effect of TCS_s_ dominates and the resulting high streamline density leads to microcarrier interactions that cause cell damage. However, the collision frequency depends on the local particle concentration. The main function of the impeller is to suspend the microcarriers by applying a lift force to oppose the gravitational force that would otherwise make them settle. During this process, the kinetic energy from the impeller is transferred to the microcarrier as potential energy. Microcarriers gain more potential energy the closer they are to the liquid surface, and this energy is converted into kinetic energy during particle sedimentation. If the impeller introduces sufficient energy into the system to keep the particles in suspension but no surplus, a homogeneous flow pattern arises. However, excess energy accelerates the particles and establishes a speed gradient in the vessel, which causes higher shear stress. For shear-sensitive suspensions that consist of solid and liquid fractions, the impeller should therefore be set at the minimum speed needed to keep the particles in suspension, and this minimum agitation rate should also be used as a scale-up criterion (Nienow, 1997, 2006). However, the models used to determine minimal agitation rates are not transferable between different bioreactors so the optimal mixing characteristics of each system must be determined empirically. The intersection of TCS_ε_ and TSC_s_ for the corresponding particle sizes provides a good approximation for this minimal agitation rate. The calculation of the average TCS revealed that energy dissipation to the cells via Kolmogorov eddies (TCS_ε_) dominates at shear stress levels below 0.13 N m^−2^, whereas energy dissipation to the cells via particle collisions in streamlines (TCS_s_) dominates at shear stress levels >0.22 N m^−2^. Our results fit well with this model: all particles were suspended at shear stress levels of >0.1 N m^−2^, the maximum virus titers were achieved at 0.1 N m^−2^, and higher shear stress (≥0.25 N m^−2^) reduced the virus titer. We therefore propose that a critical process parameter for Measles virus production is the minimal power input required to ensure all particles remain in suspension.

Agitator-dependent shear stress is not the only component of STR fermentation that causes cell damage. We found that aeration also had a dramatic effect on Measles virus production. The cell-damaging effects of gassing include the release of bubbles from the gassing tube, the interaction of bubbles with cells and carrier materials, foam formation, the coalescence and bursting of bubbles in the liquid, and the bursting of bubbles on the surface of the medium (Chisti, 2000; Flickinger and Chisti, 2009; Flickinger et al., 2010). In small-scale processes (laboratory and pilot scale) bubble coalescence and the release of air bubbles from the gas supply pipe have a minimal impact due to the low reactor height and the low volume flows, but the bursting of bubbles at the surface has been identified as a key factor (Michaels et al., 1996). If a gas bubble bursts when it reaches the liquid surface, the liquid film drains from the surface of the cavity under the force of gravity. The rupture of a gas bubble therefore accelerates the fluid flow along the bubble cavity until the lowest point of the bubble is reached, whereupon the energy is converted into a vertical fluid jet into the medium. The resulting localized shear stress is highly damaging to cells: in a culture with an average cell viability of 90%, the viability of cells directly under a bursting gas bubble is reduced to only 5% (Garcia-Briones et al., 1994; Trinh et al., 1994). Protective additives such as Pluronic F68 reduce this shear-related cell damage but also limit the oxygen transfer rate (Murhammer and Pfalzgraf, 1992). A higher agitation rate would therefore be required to ensure a sufficient oxygen supply, which would increase the shear stress and negate the positive effect of the additive. Fetal bovine serum is also known to protect cells against shear stress (Chisti, 2000). Cells growing in serum-free medium therefore tend to be more shear sensitive and require additives such as Pluronic F-68 or albumin (van der Pol and Tramper, 1998; van der Valk et al., 2010). Interestingly, in our STR environment, we observed almost no difference in the growth or productivity of Vero cells in serum-containing vs. serum-free medium at low and moderate shear rates, and the protective effect of the serum was only evident at the highest shear stress level we tested. However, high virus titers could only be achieved at low cell concentrations with head-space aeration, and the latter is not a practical aeration strategy for large-scale Measles virus production. Bubble-free aeration should therefore be established, which we previously found to be suitable for cell cultures with a high oxygen demand (Zitzmann et al., 2017). Our data make it clear that efficient virus production is only possible within narrow process windows, regardless of the cell line used as a production host.

Infection with the Measles virus triggers major changes in glycolysis and lipid metabolism, and modulates various signaling pathways and transcription factors, which means that the production host must be selected carefully to ensure that such changes to not reduce cell viability (Sato et al., 2008; Li et al., 2015). We have shown that, in addition to these intrinsic requirements, the performance of the production host is critically dependent on two major parameters—the shear stress caused by agitation and the shear stress caused by aeration. The modification or scale-up of Measles virus production must therefore take these critical process parameters into account to ensure that cell viability and high virus titers are maintained.

## Conclusion

Measles virus can be produced at high titers in Vero cells but this requires an intricate understanding of the critical process parameters to ensure that high titers can be achieved in a reproducible manner. We have shown that shear sensitivity is a key determinant of the productivity of the host cells, and that factors that induce shear stress—principally agitation and aeration—must be accommodated into any process development or process optimization strategy. Measles virus production can be defined as shear sensitive given the calculated inactivation rate of more than −8.3 log_10_ TCID_50_ N^−1^ m^2^. In order to ensure high virus yields when transferring the process to different facilities or different scales, narrow process limits must be observed to maintain optimal shear stress levels (0.1 N m^−2^). If these parameters can be achieved, it should be possible to produce virus titers of up to ~10^10^ TCID_50_ mL^−1^ in both serum-containing and serum-free medium in a reproducible manner.

## Author Contributions

TG designed and did the experiments. She also wrote the paper. DL and HD assisted in the conduct of the experiments. TW, DS, and PC helped to draft and revise the manuscript, and supervised the research. All authors contributed to manuscript revision, read and approved the submitted version.

### Conflict of Interest Statement

The authors declare that the research was conducted in the absence of any commercial or financial relationships that could be construed as a potential conflict of interest.
