# Supplementary material for: Aeration and Shear Stress Are Critical Process Parameters for the Production of Oncolytic Measles Virus
Source: Front Bioeng Biotechnol. 2019 Apr 17;7:78. doi: 10.3389/fbioe.2019.00078 (PMC6478815; doi:10.3389/fbioe.2019.00078)
Supplement: Supplementary file 1 [file Table_1.DOCX]

**Supplementary material**

**A1: Effect of microcarrier on turbulence / power input**

The flow regime in a STR can be defined as turbulent if the Ne-number is constant by increasing the Re, or the agitation rate, respectively.

For the characterization of the flow regime of the used STR, the power number were determined based on the power input at agitation rates in the range from 0-350 rpm. The power input was estimated by a torque (M) measurement (Visko-pakt rheo 35, Hightec Zhang) and by using following equation:

$P=M\cdot2\pi\cdot n$

The torque measurement was conducted five times by using culture medium with and without 3 g L^‑1^ Cytodex 1 microcarrier.

As indicated in figure S1, no difference in measured torque was observed for the mixing process with and without microcarrier. The power number kept constant for culture medium with or without microcarrier.

Weinspach et al. showed that the flow regime is influenced at a solid particle concentration of > 39% (v/v) [1]. We used a Cytodex 1 microcarrier suspension in a concentration of approximately 10 % (v/v). In accordance to the investigations by Weinspach et al., our own power characteristic of the used STR showed that the used microcarrier concentration had no impact on the turbulence in investigated design space.

**A2: Determination of the kla values of used STR**

The kla value was determined for the in section 2.4 defined design space and process conditions. The kla determination was conducted by applying the method of dynamic gassing-out using N_2_ gas and air. Therefore, a program was built up which allows a fully automated kla value determination. The usage of the flexible control system and the Hitex software provided by Hightec Zhang, the agitation rate as well as the O_2_ and the N_2_ valve (open, closed) could be adjusted automatically. In a first step the agitation rate was changed stepwise. After a constant agitation rate was archived, the N_2_ valve was closed and the O_2_ valve was open for aeration. If a DO_2_ Level of 5 was achieved, the agitation rate was changed again and the O_2_ valve was closed and the N_2_ valve was opened. This procedure was repeated until the minimal agitation rate was reached. As an example, for these experiments, following figure demonstrate the kla value determination at an aeration rate of 0.02tvvm.

The kla value was calculated based on the measured oxygen transfer rate in accordance to following relationship:

$$kla= \frac{OTR}{(c_{O2}^{*}- c_{O2})}$$

For the determination of the OTR the slope of the oxygen concentration in the range of 20-80 % was calculated. The determined kla values are shown in tab. S1.

**Reference**

[1] P.M. Weinspach, Stoffübergang im aufsteigenden Flüssigkeitsstrom, Chemie Ingenieur Technik 37(12) (1965) 1215-1220.

**Table**

Table S1: kla values for 1 L STR equipped with a 3x45°pitched blade impeller with a working volume of 0.5 L and an aeration rate of 0.02 vvm. The model suspension was DMEM-HG + 10% FBS and 3 g L^-1^ Cytodex 1 microcarrier

| agitation rate (rpm) | kla  (h^-1^) |
| --- | --- |
|  | aerated |
| 190 | 15.1 |
| 170 | 10.3 |
| 150 | 9.9 |
| 130 | 9.2 |
| 110 | 9.1 |
| 90 | 7.9 |
| 70 | 6.7 |
| 50 | 6.1 |

**Figures**

Figure S1: Torque measurement for the characterization of the flow regime in the used STR. (n=5)

Figure S2: Calculated power number for a 1 L STR equipped with a 3x 45° pitch blade impeller. DEMEM-HG supplemented with 10% FCS with and without 3 g L-1 Cytodex 1 microcarrier was investigated. (n=5)

Figure S3: Automated kla value determination based on dynamic gassing out. Here demonstrated for a 1 L STR (0.5 L working volume) equipped with a 3x 45° pitched blade impeller. The STR was filled with DMEM-HG supplemented with 10% FBS.
